# MIS-C, inherited metabolic diseases and methylmalonic acidemia: a case report and review of the literature

**DOI:** 10.1186/s13052-025-02052-1

**Published:** 2025-07-01

**Authors:** Maria Cristina Maggio, Cinzia Castana, Marina Caserta, Antonella Di Fiore, Vittoria Siciliano, Giovanni Corsello

**Affiliations:** 1 University Department PROMISE “G. D’Alessandro”, University of Palermo, Via del Vespro 129, Palermo, 90100 Italy; 2 Paediatric Clinic, Children Hospital “G. Di Cristina”, ARNAS Palermo, Palermo, Italy; 3 Paediatric Anaesthesia and Resuscitation, Children Hospital “G. Di Cristina”, ARNAS Palermo, Palermo, Italy

**Keywords:** MIS-C, Inherited metabolic diseases, Methylmalonic academia, Anakinra, Intravenous immunoglobulins

## Abstract

**Background:**

Methylmalonic acidemia (MMA) secondary to mutase deficiency, *mut0*, is an inborn error of metabolism causing complete enzyme defect, allowing a high risk of irreversible complications, secondary to metabolic decompensation, induced by infections and the hyperinflammatory state.

Multisystem Inflammatory Syndrome in Children (MIS-C) is a hyperinflammatory syndrome that manifests 14–60 days after the severe acute respiratory syndrome coronavirus 2 (SARS-CoV-2) infection in patients aged < 21 years. Only a few cases of patients with inherited metabolic diseases (IMD) and MIS-C are described. However, to our knowledge, this is the first case of MMA with MIS-C.

**Case presentation:**

We describe a 2-year-old child with MMA secondary to mutase deficiency, carrying the homozygous mutation c.2179 C > T of *MMUT* gene, associated to *mut0* phenotype.

One month after SARS-CoV-2 infection, he presented fever, rash, significant increase of C-reactive protein (CRP), ferritin, triglycerides, interleukin (IL)-6, N-terminal fragment of the pro brain natriuretic peptide (NT-pro-BNP), compatible with the diagnosis of MIS-C. He was treated with intravenous immunoglobulins and methylprednisolone, with rapid clinical improvement. Ten days later, he showed the worsening of clinical and hematological parameters, associated with anemia, thrombocytopenia, metabolic acidosis, hyperlactatemia, increased urinary methylmalonic acid, leading to multiorgan failure (MOF). He was treated with high caloric intake nutrition by intravenous carbohydrates infusion; sodium bicarbonate, thiamine, carnitine, coenzyme Q, vitamin C, antibiotics, methylprednisolone and anakinra. Three days after the start of anakinra, a significant improvement in clinical and biochemical parameters occurred. Twenty days later, a sepsis from Methicillin-resistant Staphylococcus Aureus and Candida Albicans required the interruption of anakinra, with the decline of the clinical conditions and the exitus.

**Conclusions:**

In patients with a severe form of MMA and MIS-C anakinra is a safe treatment. MOF and metabolic decompensation, secondary to the hyperinflammatory state typical of MIS-C, can be successfully treated with targeted therapy against proinflammatory cytokines. The description of these clinical cases is a precious lesson in managing IMD therapeutic emergencies. Paediatricians must provide a strict monitoring of metabolic compensation, to avoid irreversible complications.

## Background

Multisystem Inflammatory Syndrome in Children (MIS-C) is a hyperinflammatory syndrome characterized by fever, inflammation, multiorgan impairment that manifests 14–60 days after the severe acute respiratory syndrome coronavirus 2 (SARS-CoV-2) infection in patients aged < 21 years. The Centers for Disease Control and Prevention (CDC) case definition of MIS-C developed a new case definition for MIS-C diagnostic criteria, including: persistent fever > 38° C, without an alternative diagnosis and the exclusion of other microbiological etiologies, in a patient with a serious clinical picture; C-reactive protein (CRP) > 3 mg/dl; > 2 new-onset manifestations of the following: conjunctivitis, oral mucosal changes, rash, erythema and/or oedema of the extremities; abdominal pain, vomiting, diarrhoea; lymphocyte count < 1000/µl, platelet count < 150,000/µl. The most common clinical signs and symptoms include: myocardial dysfunction, capillary leak and cardiogenic shock; respiratory symptoms, acute kidney injury, neurologic symptoms (headache, aseptic meningitis, irritability); thrombosis, Macrophage Activation Syndrome (MAS) [1, 2]. Increased CRP, erythrocyte sedimentation rate (ESR) and interleukin (IL)−6, neutrophilic leukocytosis, lymphopenia and organ dysfunction are secondary to systemic inflammation and cytokine storm [3]. Patients with MIS-C show a significant overlap of symptoms with Kawasaki disease (KD) [4–7], but the actual difference between the two pathological conditions is still under discussion.

Patients with inherited metabolic diseases (IMD) have COVID-19 infection severity and long COVID incidence like the general population. The risk of acute metabolic decompensation is not higher than that found in other infections. However, COVID-19 severity in IMD patients may be correlated to disease category (complex molecule degradation) in children, and comorbidities in adults. In fact, in a case series of patients with 27 different IMD and COVID-19 infection, 2/131 children (1.5%) had MIS-C and and were treated with favipiravir, intravenous immunoglobulin, anti-inflammatory drugs, anakinra, antibiotics and anticoagulants. The high incidence of MIS-C may be coincidental but warrants further studies [8]. These possible associations must be further investigated, also based on the clinical practice of experts in the field. However, only a few cases of patients with IMD and MIS-C are described. To our knowledge, this is the first case of MIS-C in a patient with Methylmalonic acidemia (MMA).

Among IMD, MMA is a severe and rare condition. MMA secondary to mutase deficiency, *mut0,* is an inborn error of metabolism causing complete enzyme deficiency. This form represents the worst condition of the phenotypical spectrum of the disease, and it is characterized by the complete absence of enzyme activity. The disease is characterized by fever, recurrent ketoacidosis crises or transient vomiting, fatigue, dehydration, hypotonia, frequent infections, developmental delay, intellectual disability, hepatomegaly, chronic kidney disease, pancreatitis, cardiomyopathy, metabolic stroke, coma and death. Despite a specific dietary treatment, with the restriction of natural protein, particularly of propiogenic amino acid precursors, and the support of a high-caloric diet, patients undergo life-threatening metabolic imbalance. Further long-term complications include progressive renal failure, metabolic stroke and other neurological symptoms. Severe infections can act as a trigger.

## Case presentation

We describe the clinical case of a 2-year-old child with MMA secondary to mutase deficiency, with the documented homozygous mutation c.2179 C > T of *MMUT* gene, associated to *mut0* phenotype. He was the son of consanguineous parents. Both parents present the same heterozygous mutation. The diagnosis was made through Extended Newborn Screening (ENS), subsequently confirmed through genetic study, conducted via next generation sequencing panel. Clinical phenotype and metabolic analysis were confirmatory of the diagnosis. He was treated from the first days of life with a high-caloric diet and the restriction of natural protein, particularly of propiogenic amino acid precursors, associated with carnitine, hydroxocobalamin. However, he showed poor metabolic control, with recurrent vomiting, fatigue, dehydration, developmental delay, hepatomegaly. In fact, the little patient's therapeutic program included a liver transplant. In fact, liver transplant may be considered in patients with significant metabolic instability [9].

When he was 2-year-old, he had COVID-19 with fever associated with mild respiratory symptoms.

A month after contracting SARS-CoV-2 infection, he developed fever, rash, significant increase in CRP, ferritin, triglycerides, IL-6, N-terminal fragment of the pro brain natriuretic peptide (NT-pro-BNP), AST, ALT, gamma-GT, compatible with the diagnosis of MIS-C. ECG and echocardiography were normal and documented the absence of pericardial effusion, coronary artery lesions (CAL) and myocardial dysfunction. He was treated with intravenous immunoglobulins (2gr/Kg), methylprednisolone (2 mg/Kg/day), with rapid clinical improvement. Ten days later, he showed the worsening of clinical conditions, with the recurrence of fever, vasculitic rash with palmoplantar extension, further increase of ferritin (1,033 ug/l), IL-6 (146 pg/ml), NT-pro-BNP (5,117 pg/ml), triglycerides, associated with anemia, thrombocytopenia (25,100), metabolic acidosis (pH: 7.17; ammonia: 60 mcg/dl; BE: −8; HCO3: 13) with hyperlactatemia (180 mg/dl), along with increased urinary methylmalonic acid (2,380 mmol/mCreat), leading to multiorgan failure. Electrocardiogram and echocardiography were normal, and he did not develop CAL. He received nutrition with high caloric intake by intravenous carbohydrates infusion, stop of proteins intake in the first step, followed by an amino acid preparation excluding toxic amino acids as isoleucine, methionine, threonine, valine; sodium bicarbonate, thiamine, carnitine, coenzyme Q, vitamin C, antibiotics, methylprednisolone and anakinra (2 mg/Kg/day). Three days after the start of anakinra, he showed a significant improvement of clinical and biochemical parameters, with the resolution of fever, vasculitis, rash, and the reduction of CRP, triglycerides, NT-pro-BNP (50 pg/ml), IL-6 (8 pg/ml), ferritin (506 ug/l) (Table 1). Unfortunately, 20 days later, sepsis due to Methicillin-resistant Staphylococcus Aureus and Candida Albicans, infection documented by blood culture, required the interruption of anakinra and the treatment with antibiogram-based therapy. However, the worsening of the clinical and hematological parameters with multi-organ failure (MOF) and the exitus occurred.

**Table 1:** Hematologic parameters of the child correlated to treatment

Haematological parameters	6/05/2022	since 16/05/2022	since 26/05	05/06/2022
Treatment	admittance	Improvement after mPDN (2 mg/kg/die and IVIG)	Worsening of clinical conditions	Improvement after mPDN + anakinra
Hb (g/dl)	6.4	7.8	6.4	10.3
WBC/µl	1.96	9.34	5.81	9.68
Neu/lymph %	37.4/57.2	28.1/66.8	29.2/65.1	15.7/74.2
platelet/µl	88,300	45,300	25,100	102,200
CRP (n.v. < 0.5 mg/dl)	20.33	0.06	30.61	0.68
IL-6 (pg/ml)	97	11	146	8
Procalcitonin (n.v. 0.02–0.05 µg/L)	50.71	14.89	1.71	0.9
Protein/albumin (g/dl)	4.1/2.60	5/3.70	4.8/3.7	5.2/3.9
Creatinine (mg/dL)	0.18	0.30	0.23	0.17
Na/K/Cl (mmol/l)	133/4.61/98	135/3.82/100	142/3.89/105	145/3.69/102.2
Mg/P/Ca (mg/dl)	1.9/3.9/8.6	1.76/2.7/8.9	1.95/3.6/9.9	1.76/4.7/9.8
AST/ALT (UI/l)	238/112	42/83	155/93	86/55
CPK/LDH (UI/l)	32/315	23/218	36/232	/319
Gamma-GT (UI/l)	471	89	547	544
Troponine (ng/L)	9.4	5.7	42.7	15.7
NT-pro-BNP (pg/ml)	936	903	5117	50
INR/APTT	1.31/31.9	1.16/23.9	1.19/77.4	1.01/21.3
D-Dimer (ng/ml)	1.12	0.60	1.49	
Fibrinogen mg/dl	137.3	88	98.6	182.5
Ferritin (n.v. 10–232 ng/ml)	500.9	638	1,033	506
triglycerides (mg/dL)	245	523	830	51
CK-MB (n.v	2.2	2	1.5	1.5
Myoglobin (n.v. 25–58 ng/ml)	23	42	32	40
Ammonemia (n.v. 27–102 ug/dl)	46		74	
methylmalonic acid (n.v. < 7 mmol/mCreat)	5,400	199	2,380	1,280

## Discussion and conclusions

In a large case–control study, including MIS-C cases and SARS-CoV-2-positive outpatient controls less than 18 years old, metabolic or confirmed or suspected genetic disorders were not associated with a higher risk to develop MIS-C [10]. Furthermore, patients with MMA do not have a theoretical risk of developing MIS-C, as, moreover, is indirectly evident from the international literature. In fact, this is the first case described with this association.

However, patients with IMD, who frequently experience multiorgan dysfunctions, may be at risk of acute or chronic metabolic decompensation. In these patients an infection may trigger life-threatening episodes. The expected risk of severe COVID-19 infection is greater in children with disorders of complex molecule degradation, many of which are lysosomal storage diseases (LSD) often characterized by progressive multisystem involvement [11].

At the beginning of the pandemic, rare disease specialists were troubled about the impact of SARS-CoV-2 infection in these patients. In a survey, all healthcare providers described 13 paediatric cases of COVID-19 [12].

In a large cohort of patients with IMD, 6 patients (2.7%) had moderate or severe COVID-19, two (0.9%) patients died for a lethal COVID-19. Three patients (one with 3-hydroxy-3-methylglutaryl-coenzyme A [HMG-CoA] lyase deficiency, one with long-chain 3-hydroxyacyl-CoA dehydrogenase [LCHAD] deficiency, and one with MMA) had an acute metabolic decompensation during the infection and were hospitalized. Standard treatment protocols for metabolic decompensation were applied. However, they did not develop MIS-C. Two children (1.5% of the pediatric patients) (one with LCHAD and the other with biotinidase deficiency) developed MIS-C and recovered with treatment with favipiravir, IVIG, anakinra, anti-inflammatory drugs, antibiotics, anticoagulants [8]. The high incidence of MIS-C in this cohort may be casual but needs further studies [8].

In a retrospective observational study conducted in Poland, almost all patients with infection reported mild symptoms, typically described in COVID-19. Only in three children, the worsening of primary disease symptoms was observed: two patients with Niemann Pick type C (NPC) and one with Niemann Pick disease type B (NPB). They showed psychomotor agitation or motor skills worsening, despite the maintenance of symptomatic treatment during SARS-CoV-2 infection.

One pediatric patient with very long-chain acyl-CoA dehydrogenase deficiency (VLADD) was hospitalized because of metabolic decompensation. One patient with Fabry disease had an ischemic stroke, secondary to thromboembolic complications. Fabry disease, in fact, predisposes endothelial dysfunction with secondary hemorrhagic or ischemic events [13]. Furthermore, the pathogenetic mechanisms that predispose endothelial damage are attributable to chronic inflammation. These characteristics of the pathology are fertile ground for the onset of vascular events during MIS-C, typically characterized by cytokine storm and thromboembolism [14–17]. Furthermore, COVID-19 can also induce the onset of vasculitis not associated with a clinical picture of MIS-C [16]. In these patients, a genetic background such as Fabry disease, MELAS or hyperhomocysteinemia [18], can predispose to this fearful complication. However, the risk of vasculitis in pediatric age is greater in patients with a clinical picture of MIS-C.

A study describing the impact of SARS-CoV-2 infection on metabolic outcome in IMD patients, showed that the most frequent clinical signs were fever (52.1%) and fatigue/myalgia (47.8%). None of the patients presented severe or critical disease. However, four patients (two patients diagnosed with propionic acidemia, one patient with methylmalonic acidemia and one patient with 3‐hydroxy‐3‐methylglutaryl‐CoA lyase deficiency (HMG‐CoA lyase deficiency)) developed a metabolic decompensation, with clinical and biochemical findings of an acute metabolic attack. The patient with MMA had severe metabolic acidosis, hyperlactatemia, renal function disorder, during a mild COVID‐19 infection [19]. A patient with HMG‐CoA lyase deficiency had metabolic acidosis, hyperlactatemia, hypoglycemia, vomiting and insufficient feeding, during a moderate COVID‐19 infection. In these four patients, a prompt emergency treatment was performed for metabolic decompensation. In organic acidemia, patients including MMA and propionic acidemia, oral treatment was substituted by intravenous carnitine. Parenteral nutrition with hypercaloric support, lipid and glucose in addition to continuous intravenous insulin infusion was started to raise anabolism. Intravenous bicarbonate replacement was added to control metabolic acidosis, and protein intake was stopped for 24 h. The treatment of the patient with HMG‐CoA lyase deficiency included intravenous carnitine, intravenous bicarbonate and high glucose rated intravenous nutrition [19]. One case report of a 1‐year‐old patient with propionic acidemia, had a moderate COVID‐19 infection with only a slight hyperammonemia and no major changes in blood gas analysis and plasma lactate level. COVID-19 caused his first metabolic crisis. However, he recovered without a severe clinical outcome [20] (Table 2).

**Table 2:** Patients reported in literature with IMD and COVID-19 with related complications

Reference	Patients	Diseases	Outcome
Kahraman AB, et al. [8]	223	6 IMD 3 (1 with HMG-CoA lyase deficiency, 1 with LCHAD deficiency, 1 with MMA) 2 IMD (1 with Tyrosinemia type I, 1 with PKU) 2 (1 with LCHAD, 1 with biotinidase deficiency)	Moderate or severe COVID-19 Hospitalization for metabolic decompensation Died with COVID-19 MIS-C
Lampe C, et al. [11]	13		Mild COVID-19
Tobór-Świętek E, et al. [12]	44	2 with NPB and NPC; 1 with VLADD 1 with Gaucher disease type I 1 with Fabry	Mild COVID-19 Metabolic decompensation massive deep vein thrombosis ischemic stroke
Zubarioglu T, et al. [18]	22	4 patients (2 with propionic acidemia, 1with methylmalonic acidemia, 1with HMG‐CoA lyase deficiency)	Mild COVID-19 metabolic decompensation
Caciotti A, et al. [19]	1	1 with propionic acidemia	Mild COVID-19

Considering all categories of IMD, individuals with intoxication‐type metabolic disorders and energy metabolism disorders are mainly at risk for metabolic decompensation during an intercurrent infectious disease [19]. In intoxication‐type IMD, infectious diseases can trigger catabolism, which leads to endogenous breakdown of proteins and increased deposit of toxic metabolites. In energy metabolism disorders, infectious diseases can trigger catabolism that raises cellular energy request. The lack of response to this energy need leads to an energy deficit and metabolic decompensation.

Lastly, IMD do not appear to worsen COVID‐19 course. However, COVID‐19 infection can trigger a severe life‐threatening metabolic decompensation in these patients [19]. The published data of the European Registry and Network for intoxication type metabolic diseases Consortium (E-IMD), including MMA, showed that the survival rate was 100% [21].

The unfortunate outcome of our patient is linked to multiple cofactors that intervened, also based on the increased risk of severe infections and metabolic decompensation after infections in patients with IMD, although MIS-C was promptly treated with effective therapy. This case highlights the role of anakinra in a child with a severe form of MMA and MIS-C, with the significant clinical and biochemical improvement and the resolution of MOF, secondary to the cytokine storm of MIS-C and the metabolic imbalance. Furthermore, MMA is a metabolic disorder characterized by metabolic crises and the catabolic effects of glucocorticoids can exacerbate the catabolic state in MMA patients, further disrupting their metabolism, leading to an increased risk of acute decompensation. The breakdown of muscle tissue due to catabolic effects of glucocorticoids can trigger complications like muscle weakness and severe muscle damage. Due to these risks, the use of systemic glucocorticoids in MMA patients is generally avoided, particularly outside of emergency situations. However, anakinra did not improve the metabolic imbalance, as persistent high levels of methylmalonic acid demonstrate. Anakinra showed a good safety profile also in severe metabolic disease. The good safety profile is well demonstrated in international literature, in patients with systemic juvenile idiopathic arthritis [22, 23], autoinflammatory diseases [24–27] and KD [28]. Clinical data support the employment of anakinra as a first-line biologic as early as possible, to abate the cytokine cascade not exclusively in severe and/or complicated MIS-C [15, 16, 29, 30].

To our knowledge, this is the first case described in the literature of MIS-C in a child affected by MMA, treated with anakinra. Our child, as described in children with IMD, who frequently experience multiorgan dysfunctions, underwent acute metabolic decompensation during MIS-C. In our patient SARS-CoV-2 infection triggered the life-threatening cytokine storm. Hyperinflammation is an overstated metabolic stress also for children without IMD, requiring a prompt treatment with high doses glucocorticoids and biological drugs, as IL-1 antagonists (14; 16). In fact, these patients need to provide emergency treatment during acute decompensation events, with the goal of averting catabolism and minimizing central nervous system damage. Furthermore, in the table the levels of methylmalonic acid of our patient are reported. We showed that methylmalonic acid levels were extremely high during the acute phase of MIS-C, however, they significantly decreased after starting immunomodulatory therapy. This obtained goal demonstrates that the cytokine storm, the main pathogenetic basis of MIS-C, must be promptly controlled in patients with IMD, to prevent irreversible metabolic decompensation and MOF. High doses of glucocorticoids and anakinra work together to reach this target. Then again, nutrition with high caloric intake by intravenous carbohydrates infusion, stop of proteins intake in the first step, followed by an amino acid preparation excluding toxic amino acids as isoleucine, methionine, threonine, valine; sodium bicarbonate, thiamine, carnitine, coenzyme Q, vitamin C, antibiotics are the basis of the metabolic control in acute illness.

With the advent of biologic disease-modifying anti-rheumatic drugs in rheumatic diseases, children are expected to increase the frequency of common infections and the risk of serious and opportunistic infections. In a multicentric study, the “Pharmacovigilance in Juvenile Idiopathic Arthritis patients” (Pharmachild), a significant opportunistic infection rate was documented, especially for herpes simplex, tuberculosis and Candida Albicans [31]. In our child Candida Albicans and Methicillin-resistant Staphylococcus Aureus sepsis occurred and required the withdrawal of anakinra treatment.

Further studies are needed to define the appropriateness and safety of therapy with anakinra in Candida Albicans sepsis, a lethal complication the patient suffered from prolonged venous catheterization and the high risk of infections, due to the underlying metabolic disease. Finally, no less important is the need for adequate genetic counseling for families of children with IMD, with adequate information on the risk of recurrence and the medium and long-term prognosis [32, 33].

Severe infections, such as COVID-19, can act as a trigger in patients with IMD. Moreover, SARS-CoV-2 infection can induce MIS-C, a further factor triggering metabolic decompensation of these children. Despite a specific dietary treatment, patients with IMD can undergo life-threatening metabolic imbalance. Further long-term complications include progressive renal failure, metabolic stroke and other neurological symptoms.

Only a few cases of patients with IMD and MIS-C are described. However, the description of these clinical cases is a valuable lesson for paediatricians who find themselves managing such therapeutic emergencies. We must, however, consider that immuno-naïve patients, such as the patient described, may go on to more severe SARS-CoV-2 infection, with a higher risk of metabolic decompensation, despite employed treatment. Therefore, it is a key message to emphasize that preventive measures such as hygiene, social distancing, and vaccination should be considered as part of standard care in patients with IMD and recommended as in any other infectious disease (e.g.,

influenza). Anakinra should, however, be considered a safe treatment of choice in patients with IMD and MIS-C. Timely use of anakinra therapy in patients with a severe form of MMA is safe and can be employed to treat MIS-C, achieving substantial clinical and biochemical improvement. Therefore, resolution of MOF, secondary to cytokine storm, typical of MIS-C, and the ability to stem metabolic decompensation with appropriate nutritional support can be better achieved with the concomitant use of anakinra.

We emphasize that patients with MMA are extremely complex to manage and have a very high and not always predictable risk of acute metabolic decompensation. Therefore, it is necessary that they are managed at specialized centres with great expertise in the field of IMD, because the risk of death is very high in case of severe infections.

## Data Availability

The datasets used and/or analysed during the current study are available from the corresponding author on reasonable request.

## Abbreviations

MMA: Methylmalonic academia
MIS-C: Multisystem Inflammatory Syndrome in Children
SARS-CoV-2: Severe acute respiratory syndrome coronavirus 2
IMD: Inherited metabolic diseases
CRP: C-reactive protein
IL: Interleukin -6
NT-pro-BNP: N-terminal fragment of the pro brain natriuretic peptide
MOF: Multiorgan failure
CDC: Disease Control and Prevention
ESR: Erythrocyte sedimentation rate
KD: Kawasaki Disease
ENS: Extended Newborn Screening
CAL: Coronary artery lesions
LSD: Lysosomal storage diseases
HMG-CoA: 3-Hydroxy-3-methylglutaryl-coenzyme A lyase deficiency
LCHAD: Long-chain 3-hydroxyacyl-CoA dehydrogenase deficiency
NPC: Niemann Pick type C
NPB: Niemann Pick disease type B
VLADD: Very long-chain acyl-CoA dehydrogenase deficiency
HMG‐CoA lyase deficiency: 3‐Hydroxy‐3‐methylglutaryl‐CoA lyase deficiency
IVIG: Intravenous immunoglobulins
WHO: World Health Organization
